# Why a diaminopyrrolic tripodal receptor binds mannosides in acetonitrile but not in water?

**DOI:** 10.3762/bjoc.10.156

**Published:** 2014-07-03

**Authors:** Diogo Vila-Viçosa, Oscar Francesconi, Miguel Machuqueiro

**Affiliations:** 1 Centro de Química e Bioquímica, Departamento de Química e Bioquímica, Faculdade de Ciências, Universidade de Lisboa, 1749-016 Lisboa, Portugal, Phone: +351-21-7500112. Fax: +351-21-7500088; 2 Dipartimento di Chimica, Università di Firenze, Polo Scientifico e Tecnológico, 50019 Sesto Fiorentino, Firenze, Italy

**Keywords:** conformational analysis, constant-pH MD, mannose, multivalent glycosystems, pH, synthetic receptor

## Abstract

Intermolecular interactions involving carbohydrates and their natural receptors play important roles in several biological processes. The development of synthetic receptors is very useful to study these recognition processes. Recently, it was synthetized a diaminopyrrolic tripodal receptor that is selective for mannosides, which are obtained from mannose, a sugar with significant relevance in living systems. However, this receptor is significantly more active in acetonitrile than in water. In this work, we performed several molecular dynamics and constant-pH molecular dynamics simulations in acetonitrile and water to evaluate the conformational space of the receptor and to understand the molecular detail of the receptor–mannoside interaction. The protonation states sampled by the receptor show that the positive charges are always as distant as possible in order to avoid large intramolecular repulsions. Moreover, the conformational space of the receptor is very similar in water above pH 4.0 and in acetonitrile. From the simulations with the mannoside, we observe that the interactions are more specific in acetonitrile (mainly hydrogen bonds) than in water (mainly hydrophobic). Our results suggest that the readiness of the receptor to bind mannoside is not significantly affected in water (above pH 4.0). Probably, the hydrogen bond network that is formed in acetonitrile (which is weaker in water) is the main reason for the higher activity in this solvent. This work also presents a new implementation of the stochastic titration constant-pH molecular dynamics method to a synthetic receptor of sugars and attests its ability to describe the protonation/conformation coupling in these molecules.

## Introduction

The recognition of specific carbohydrates is an important step in several biological processes [1]. To better understand these recognition processes, several synthetic receptors have been developed over the years [1–6]. Most of these were developed for glucose since it is one of the most common sugars in living systems [7] and the preferred monosaccharide for energy storage [8]. However, mannose is essential for various biological functions, such as molecular recognition, being one of the most abundant sugars in glycoconjugates [9–10].

**Figure 1:**
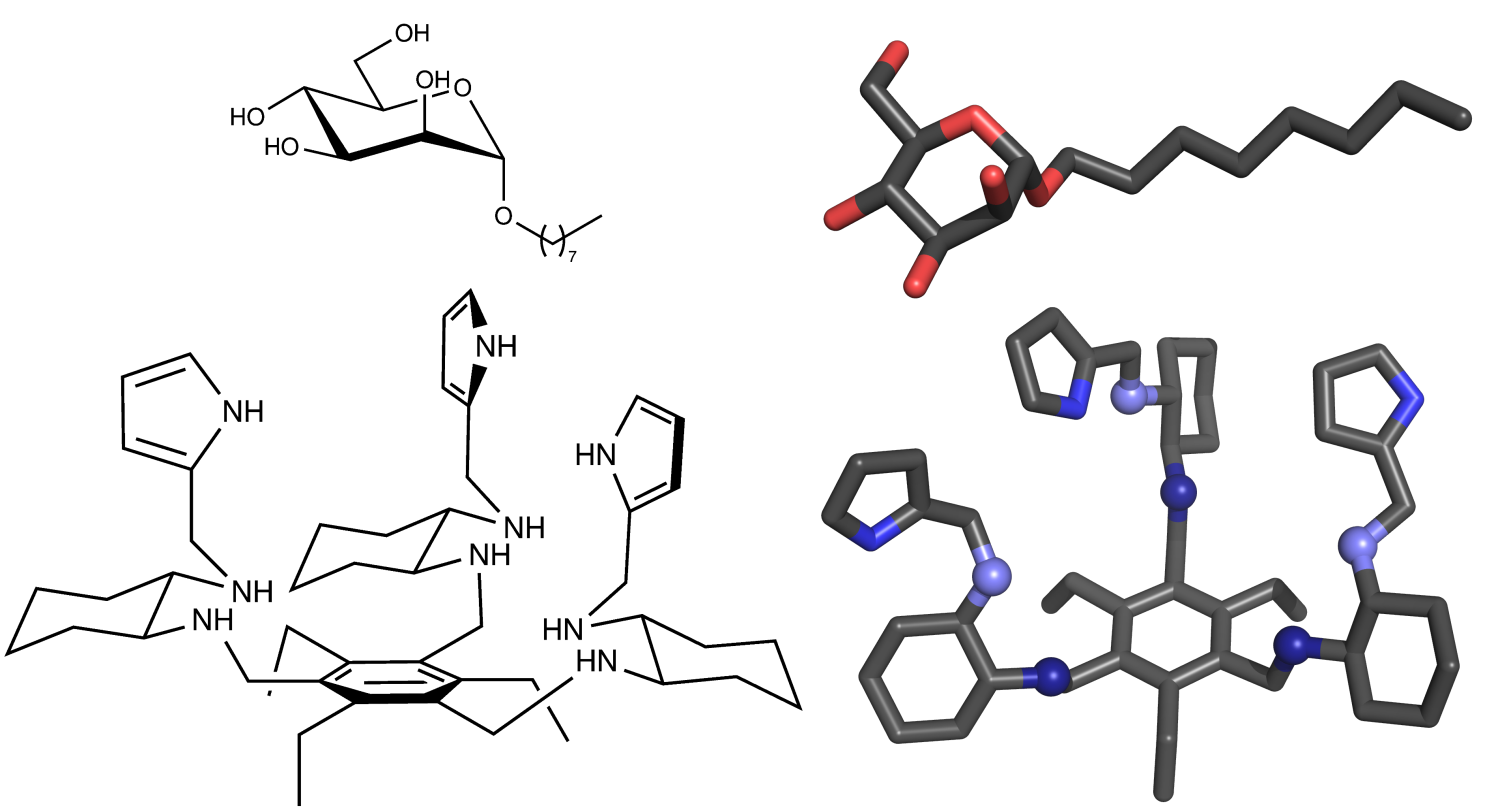
Octyl α-D-mannoside (mannoside) and diaminopyrrolic tripodal receptor molecules. 2D and 3D representations are shown. In 3D, all hydrogen atoms were omitted for clarity, and titrable amino groups are shown in spheres with first generation in dark blue and second in light blue.

In 2011, Roelens’ group [11–13] synthetized and tested a chiral diaminopyrrolic tripodal receptor that showed high binding affinities to mannosides (Figure 1). This particular receptor is significantly more active in acetonitrile (ACN) than in water [14]. In fact, this receptor family [11–13] could only be tested in water at slightly acidic conditions, due to solubility reasons, and proved to be very inefficient [14]. Unraveling the molecular details of these host–guest interactions is crucial to understand their different performances when changing the solvent and to identify the molecular determinants behind the reported high affinities.

Simulation methods, namely the so-called constant-pH molecular dynamics (CpHMD) methods, have been used to understand the molecular detail of several phenomena in the last years [15–24]. Since MD simulations deal with pH in a limited way, simply by setting a fixed protonation state in the beginning of the simulation, the use of one of these methods is mandatory. The stochastic titration constant-pH MD method [15,21] takes advantage from the complementarity between molecular mechanics/molecular dynamics (MM/MD) simulations, that correctly samples the conformational space of a system according to a classic potential energy function, and Poisson–Boltzmann/Monte Carlo methods, that can efficiently treat multiple protonation equilibria on rigid structures [25–26]. This way, it is possible to deal with pH as an external parameter that is fixed in our simulations since the protonation state of the titrable groups is allowed to periodically change during the simulation capturing the coupling between protonation and conformation. This method was already successfully applied to peptides and proteins in recent years [15,21,27–36].

The interaction of sugar molecules with receptors has been studied and shown that the recognition process involves many hydrogen bonds between the two molecules [1,37]. Aqvist et al, using MM/MD simulations, showed that the major contribution to the binding energy between both glucose and galactose to a proteic receptor, came from hydrogen bonds [38]. Moreover, Lerbret et al, suggested that hydrogen bonds are strongly related with the influence of sugar molecules in lysozyme structure [39–40].

In this work, we performed an exhaustive conformational study of a chiral diaminopyrrolic tripodal receptor (Figure 1) in both water and acetonitrile. We used MM/MD simulations in the organic solvent and constant-pH MD to simulate the receptor in water at several pH values. Moreover, the interaction between the receptor and octyl α-D-mannoside (the receptor binds strongly both α and β anomers [12]) was also studied in these two solvents. These two approaches intend to address two working hypothesis: the receptor is active in acetonitrile but not in water due to the different conformational behavior in the two environments or due to specific interactions that are (un)favored when changing the solvent. This work also extends, for the first time, the use of the stochastic titration constant-pH MD method to artificial molecules, in particular to diaminopyrrolic receptors.

## Results and Discussion

### Titration curve of the receptor

**Figure 2:**
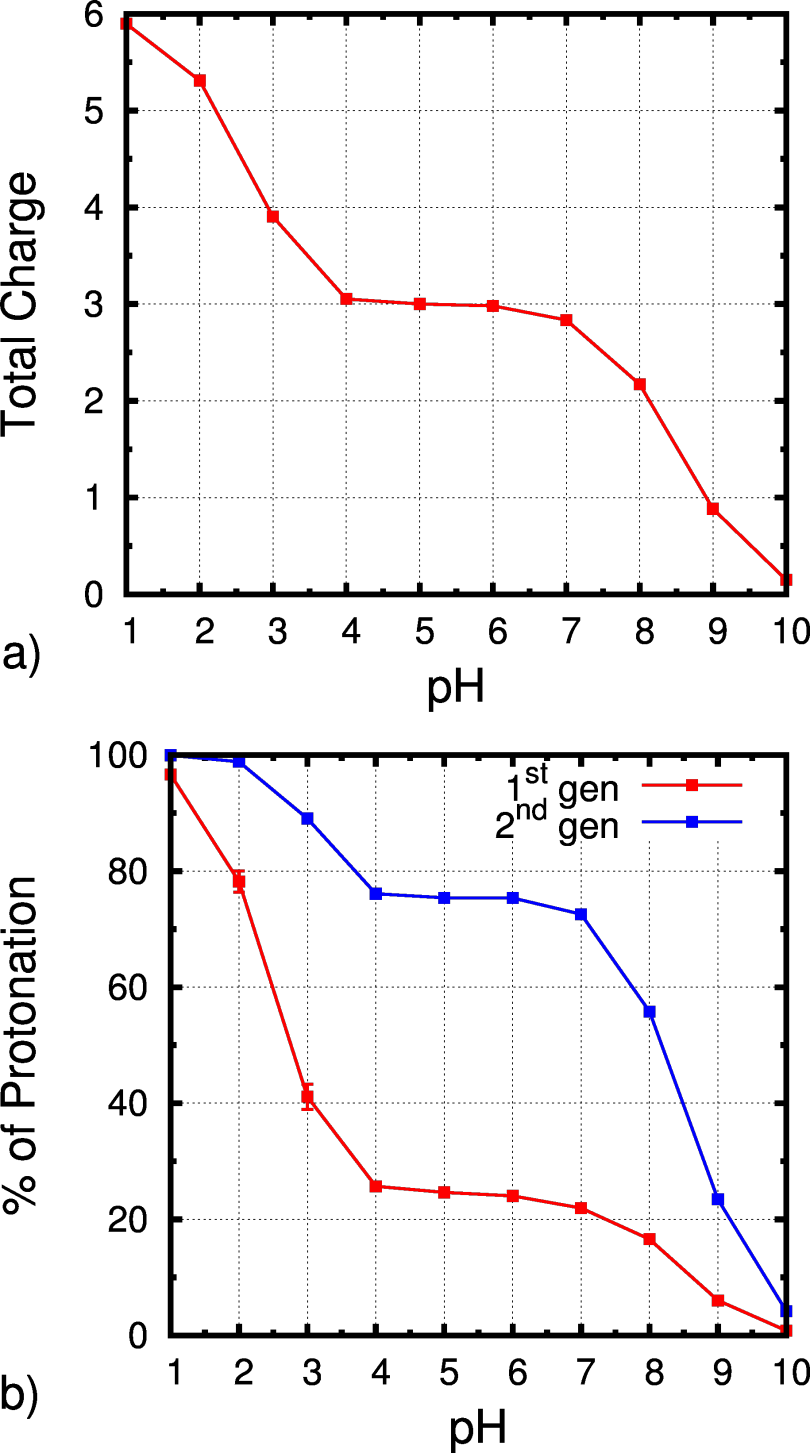
Full titration curve of the receptor (a) and the correspondent percentage of protonation in each generation (b).

The titration curve of the receptor was obtained by averaging the occupancy of all titrable amine groups at each pH value over 270 ns (last 90 ns of each replicate). The total titration curve (Figure 2a) shows a clear plateau between pH 4.0 and 7.0, approximately. In these range, the total protonation is ~3 meaning that half of the titrable groups are protonated and the total charge of the receptor is 3. At an extremely high (low) pH value the molecule is completely deprotonated (protonated) which means that the protonation range is completely sampled. From the total titration curve, we can also conclude that our receptor is only close to neutrality at pH 10.0. From the individual titration curves of the first and second generation residues (Figure 1), we can determine which of these groups are being protonated at each pH value (Figure 2b). We observe that, in the plateau between pH 4.0 and 7.0, the second generation has an average protonation of 75% and the first, 25%. The higher charge in the second generation amine groups is expected since these groups are more exposed to the solvent, interacting less with each other, which means that they are easier to ionize. In the case of the first generation amino groups, they are much closer to each other, with limited conformational freedom, resulting in much lower p*K*_a_ values.

**Figure 3:**
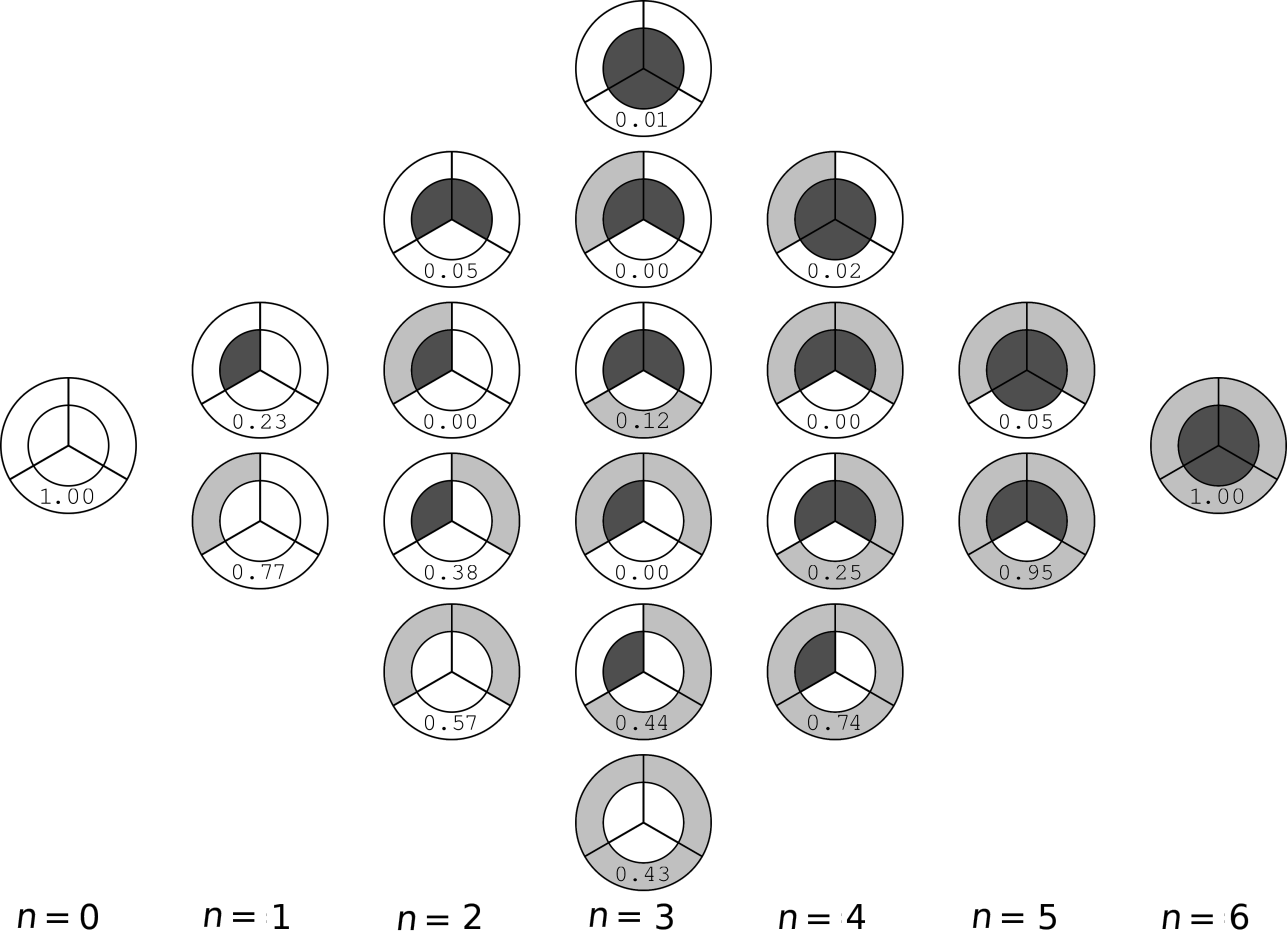
Diagram representing the population of all microstates at each number of titrable protons (*n*) present in the molecule.

The small number of titrable sites in the receptor allows us to analyze in detail the population of each possible microstate. In particular, from the populations of all simulated pH values, it is possible to draw a diagram to represent which states are more populated at each number of titrable protons present in the molecule (Figure 3). For example, when the molecule has one proton, it can be in one amino group of the first generation or in the second generation. However, if the molecule has two protons, they can be both in the first generation, both in the second generation, in different generations but in the same arm, or in different arms of the molecule (by arms we consider the diaminopyrrolic groups attached to the center phenyl group). From Figure 3, we observe that, in general, the position of protons (positive charges) is such that repulsion is minimized. For example, with two protons, the preferred state is the one with two second generation amino groups protonated which allows the two charges to be more distant to each other. Interestingly, there are many states that are almost not populated. In particular, with five protons, the state with three protons in the first generation is almost not populated since this state implies the presence of three positive charges in a very small space. With one proton, the preferred state is the one with the proton in the second generation which is more exposed to the solvent. However, since the global charge of the receptor is only one in this state, both microstates are significantly populated. With two and four protons, the observations are similar since in both cases the microstates with the second generation amino groups protonated are preferred and the ones with protons in different arms are also significantly populated. In these states, there are two microstates that are almost not populated due to the high repulsion when protonating two groups in the same arm or in the first generation. Finally, with three protons, only three (out of six) microstates are significantly populated and these are the ones that allow for smaller repulsion, as observed in the previous cases. This analysis was also performed for each pH value (see Supporting Information File 1) and the observations are completely analogous to the described above. As expected, geometrically constrained titrable sites show strong interactions due to electrostatic repulsion which results in shifted p*K*_a_ values and in some forbidden (not sampled) protonation states. These results illustrate the usefulness of the constant-pH MD methodologies and expose the strong limitations of classic MM/MD simulations to deal with such systems.

### Conformational analysis of the receptor

**Figure 4:**
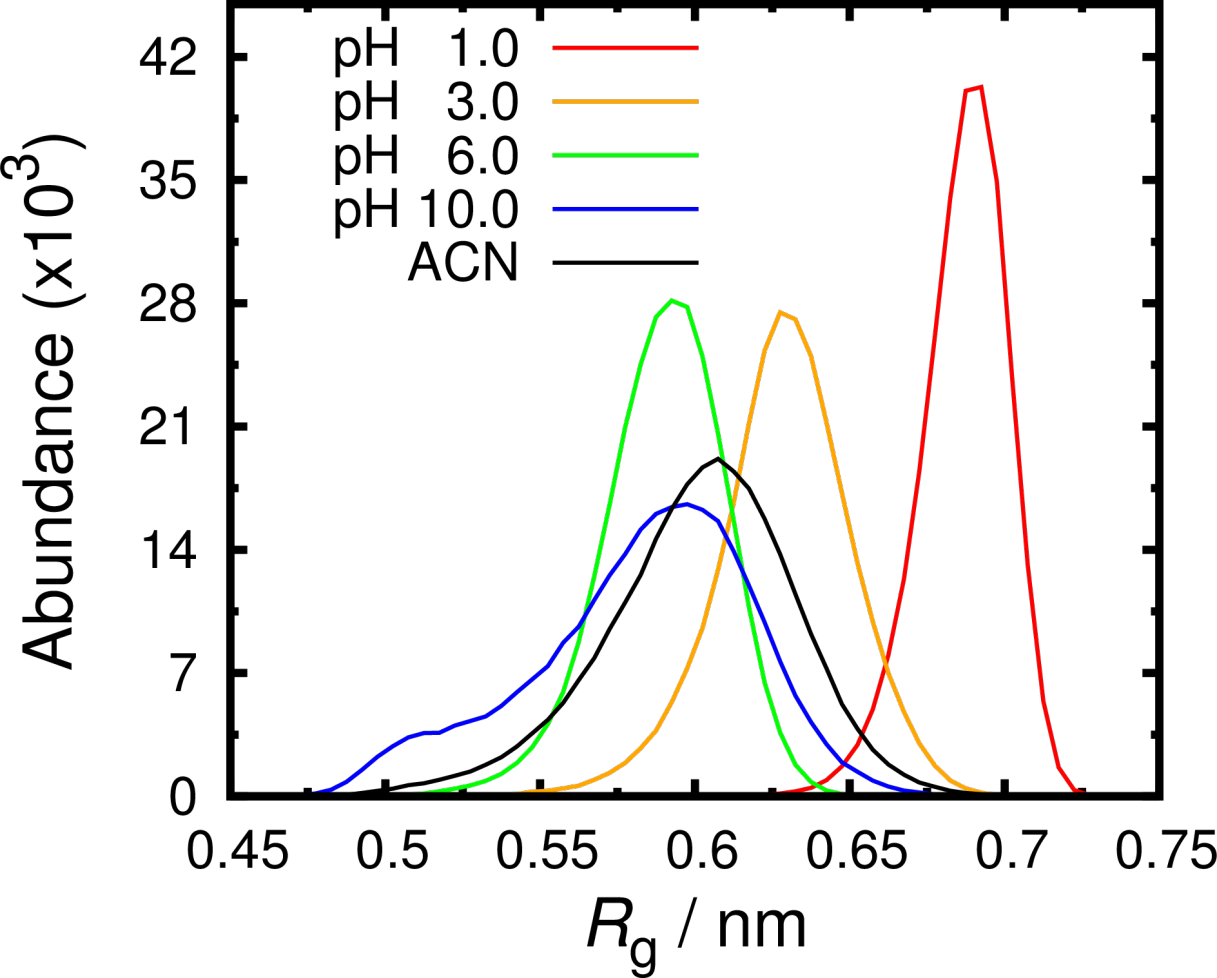
Radius of gyration (*R*_g_) histograms for the receptor in water at pH 1.0, 3.0, 6.0, 10.0 and in ACN. The *R*_g_ curves at the pH values not shown are well behaved and respect the observed trend.

As expected, both in ACN and in water at all simulated pH values, the receptor shows a large conformational variability. The radius of gyration (*R*_g_) indicates how “open” is the structure: a large *R*_g_ indicates that the receptor is in an extended conformation. Our results show that the receptor samples a large interval of *R*_g_ (Figure 4 and Supporting Information File 1). In fact, in most simulations, the molecule can sample “closed” conformations with *R*_g_ around 0.50 nm or more “open” with *R*_g_ larger than 0.65 nm. However, at lower pH values, in particular at pH 1.0, the distribution of *R*_g_ is much narrower and deviated to larger *R*_g_ values. As mentioned above, at pH 1.0, the residues are almost completely charged and this generates significant repulsion between the arms which increases the *R*_g_. At pH 10.0, the receptor is able to sample a very low *R*_g_ region (a shoulder in the histogram) that is almost inaccessible in the ACN simulations. These closed and packed conformations are favored when the receptor is neutral and is potentiated by its hydrophobicity, which does not happen in the organic solvent. Interestingly, above pH 4.0, the distribution of *R*_g_ is very similar in all simulations and comparable with the simulations in ACN. This observation suggest that the conformational space, in terms of *R*_g_, is similar in water (above pH 4.0) and in ACN. In other words, the receptor is able to accommodate up to 3 protons without any major influence in the preferred positions of its arms. This suggests that the reason hindering the receptor to bind sugars in water should not be its conformational readiness to establish specific intermolecular interactions.

**Figure 5:**
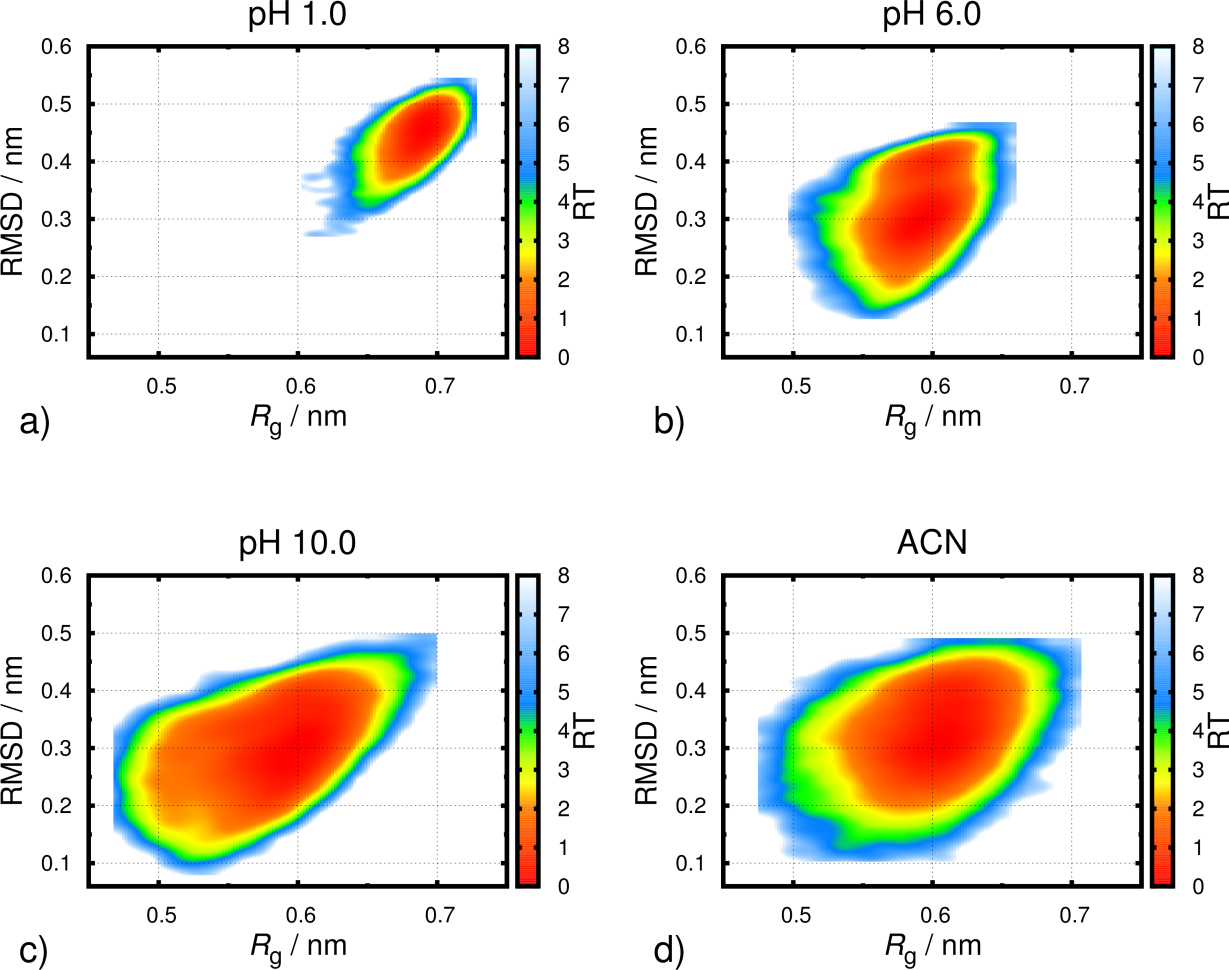
Free energy profiles for the receptor at pH 1.0 (a), 6.0 (b), 10.0 (c) and ACN (d) using RMSD and *R*_g_ as structural coordinates. The RMSD was calculated using the NMR derived structure as reference.

To further characterize the conformational behavior of the receptor in water and ACN, we combined two broadly used properties, namely *R*_g_ and RMSD, to obtain 2D-energy landscapes for each pH value and ACN (Figure 5 and Supporting Information File 1). In agreement with the *R*_g_ data, at low pH values, all sampled structures have both high *R*_g_ and RMSD. This means that, in more open conformations, the structure is also very different to the reference. Hence, in all these landscapes, we observe a correlation between RMSD and *R*_g_ properties. As the pH increases, more closed and lower RMSD structures are sampled. This is also in agreement with the *R*_g_ data which shows that the conformational space of the receptor at high pH values is similar to the one in ACN.

**Figure 6:**
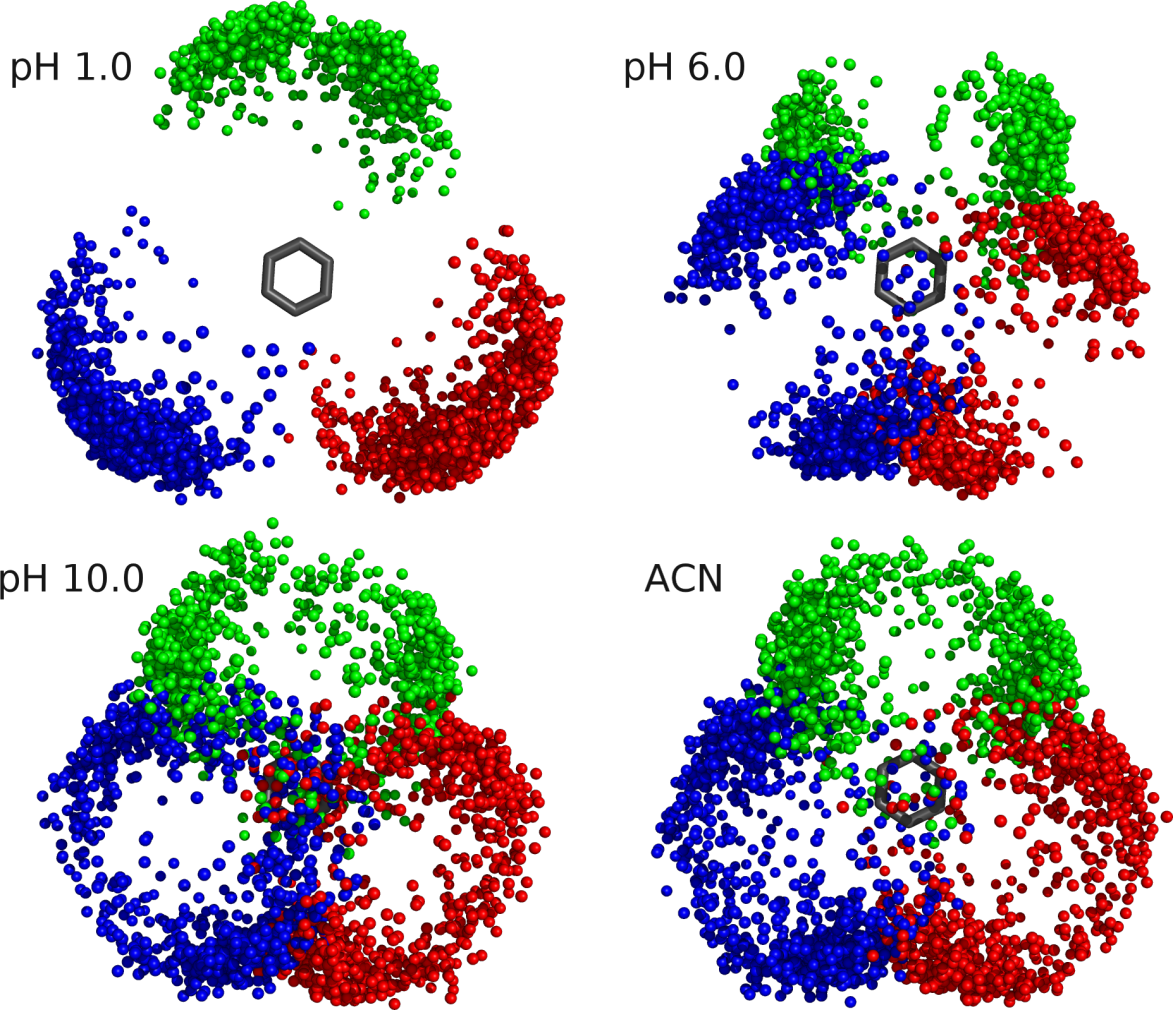
Schematic representation of the receptor arms positions. Pyrrolic nitrogen atoms positions relative to the phenyl ring in 900 conformations at pH 1.0, 6.0, 10.0 and in ACN.

Both *R*_g_ and energy landscapes data identify the position of the arms as a good descriptor of the receptor conformational space. Hence, we represented the positions of pyrrolic nitrogen atoms relative to the phenyl ring in 900 conformations (Figure 6 and Supporting Information File 1). This result illustrates that, at low pH values, the arms are very distant to each other since they are positively charged and, as pH increases, more closed structures are sampled. Moreover, as observed before, the sampled positions at high pH values are very similar to the ones in ACN.

The described conformational analyses of the receptor suggest that the behavior in water, at low protonation, is similar to the observed in ACN. This suggests that, from a conformational point of view, the receptor would be able to bind the mannoside, both in water and ACN. However, as mentioned above, it was observed that this interaction is favorable in ACN but not in water. This may be explained by the different interactions that occur in water and ACN.

### Receptor/mannoside interactions

To understand the molecular detail of the interaction between receptor and mannoside, several MM/MD simulations in water and ACN were performed without restraints (data not shown). In these simulations the residence time of the mannoside was very short (in the sub ns timescale). In addition, the binding event was never observed in our unrestrained simulations. Since entropy plays a crucial role in binding events they can be too slow and inaccessible in our computational timescale. Hence, to gain better insights into the molecular detail of the interaction between sugar and receptor, we performed the receptor + mannoside simulations (see Experimental section) with a position restraint between the phenyl group of the receptor and the sugar ring in the mannoside. This way, we were able to sample the interactions between the ligand and the receptor more efficiently and draw conclusions regarding the specificity of this event. In these simulations, we tested four protonation states (see Experimental section) from which W^q3a^ and W^q3b^ refer to the slightly acidic conditions analogous to experiments [14].

**Figure 7:**
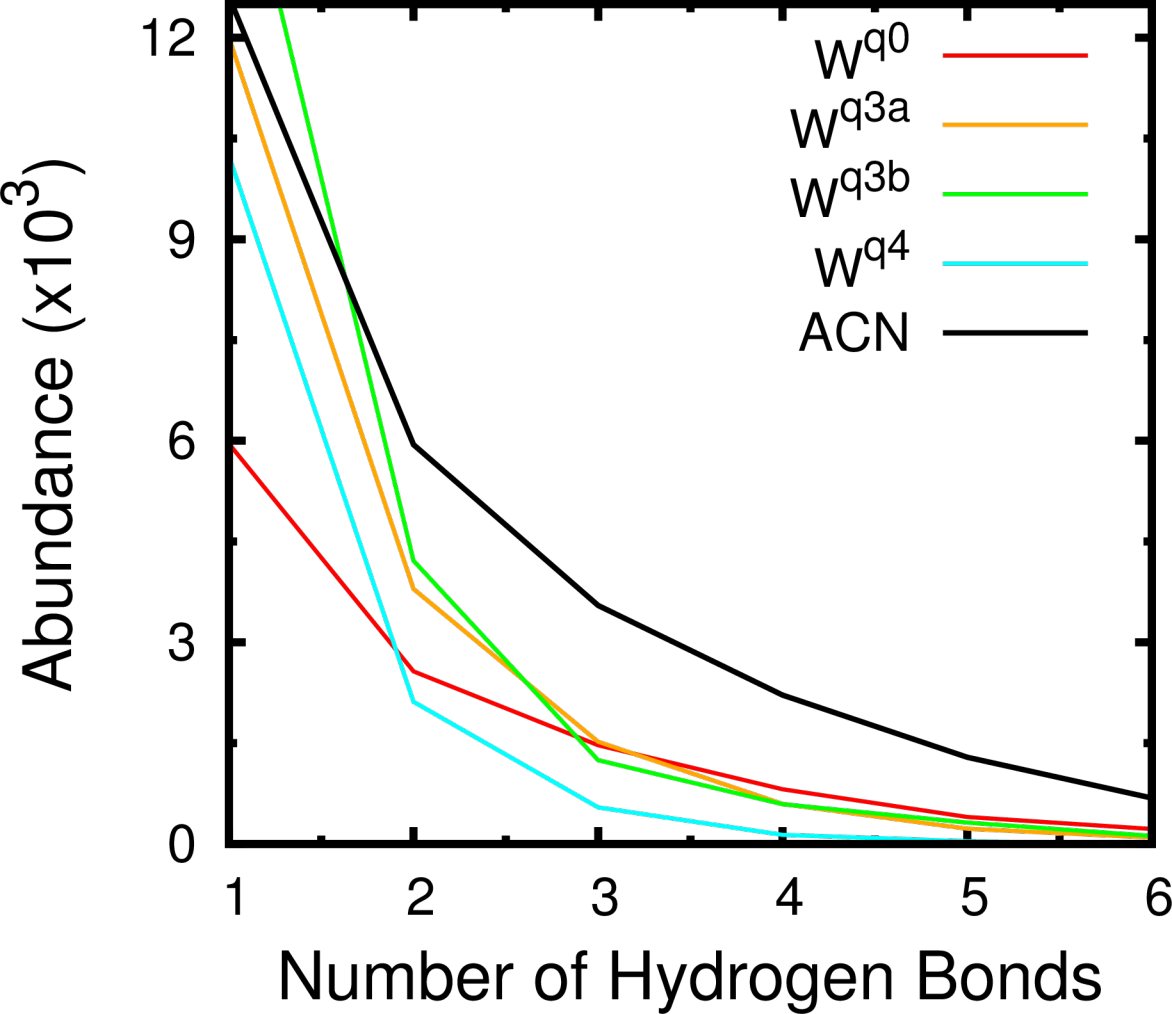
Histogram of the hydrogen bonds between receptor and mannoside in ACN and water (at different protonation states).

The histogram of the number of hydrogen bonds in ACN and water (Figure 7) suggests that in ACN the interaction is stronger. The number of conformations with more than 1 hydrogen bond is higher in ACN. Since donors and acceptors for hydrogen bonds in the mannoside are all in the head group, this result suggests that the interaction with the receptor is more specific in ACN. In water, with increased ionization, the receptor samples more open conformations favoring interactions involving only one arm (1–2 hydrogen bonds) and preventing structures with more hydrogen bonds.

**Figure 8:**
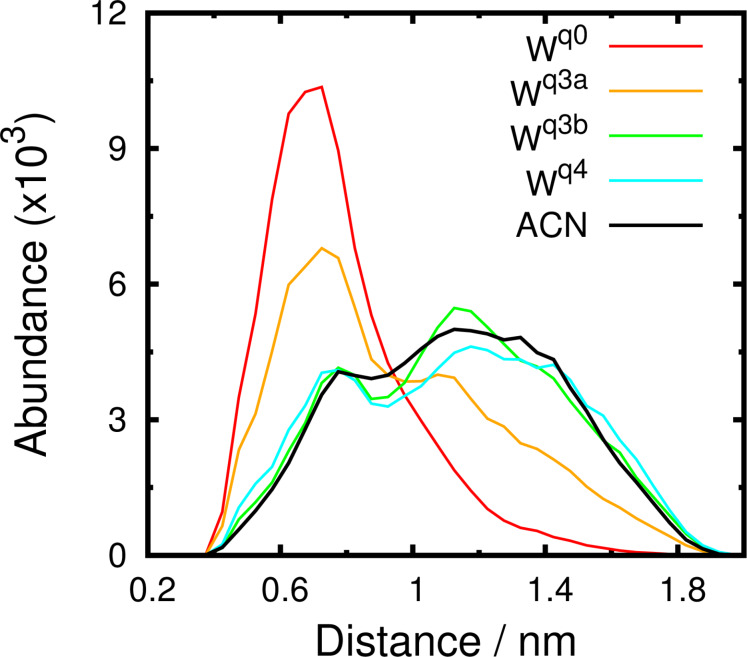
Distance histograms between the center of mass of the last 4 atoms of the carbon chain of the mannoside and the 6 atoms of the phenyl ring in the receptor. Calculations were done in ACN and water (at different protonation states).

**Figure 9:**
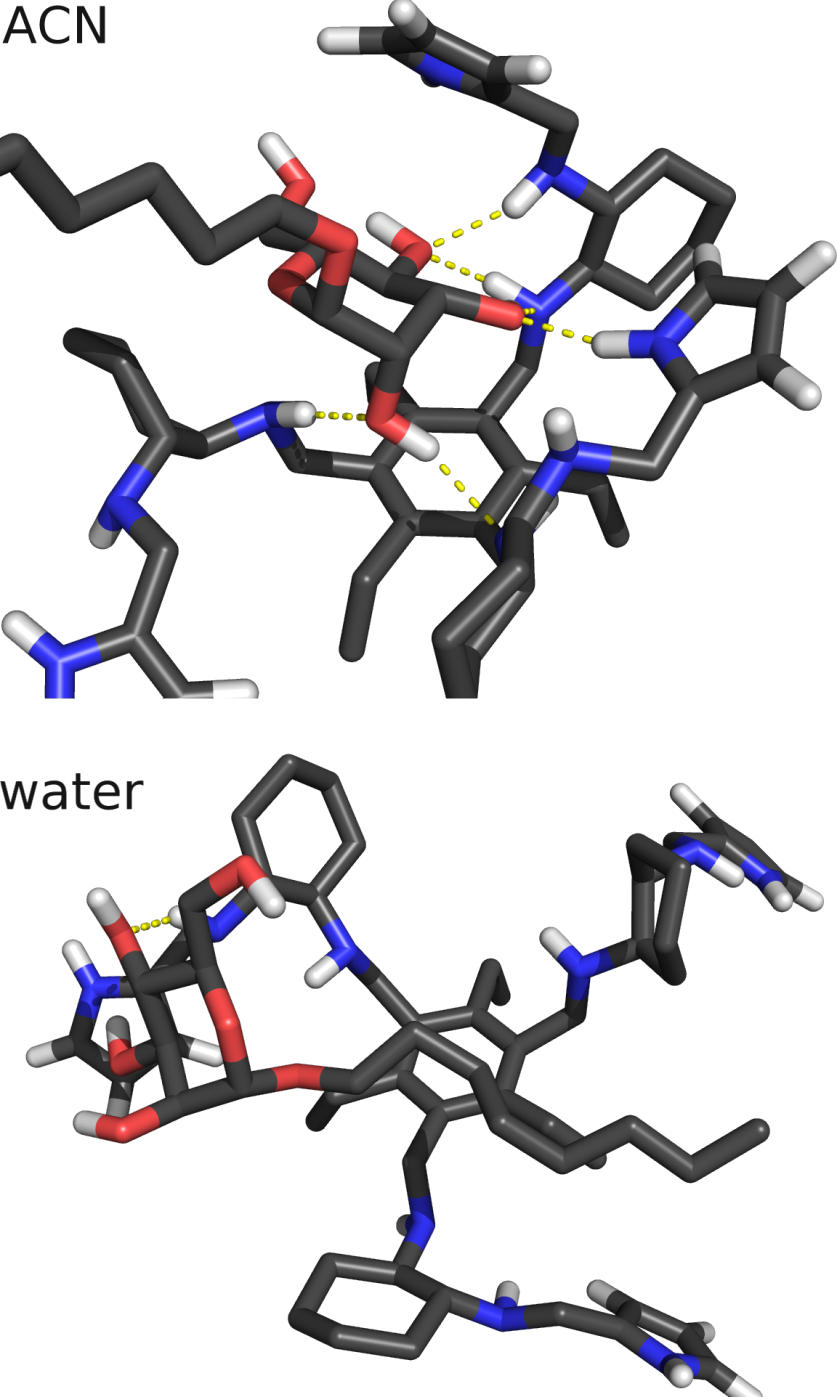
Typical conformations in water and ACN. The selected conformations have 6 hydrogen bonds in ACN and 1 in water.

We also calculated the histogram of the distance between the center of mass of the last 4 atoms of the carbon chain of the mannoside and the 6 atoms of the phenyl ring (Figure 8). These histograms show a clear preference for lower distances in water for the fully deprotonated state (W^q0^). In W^q0^, the interaction between the carbon chain and the phenyl ring is stronger and stabilized by more close conformations. The protonation of the 1^st^ generation amino groups (W^q3b^ and W^q4^) induces a higher solvent exposure which destabilizes the mentioned hydrophobic interaction. As a result, these systems behave like ACN where there is no significant interaction. These observations are illustrated with two typical conformations in W^q0^ and ACN (Figure 9). In ACN, a strong interaction between the sugar ring and the arms of the receptor is observed and, in water, the carbon chain lays close to the ring stabilized by the hydrophobic effect.

Altogether, these results show that, in ACN, hydrogen bonds are stronger and, probably, the main responsible for the intermolecular interaction. In water, hydrophobic effects may play an important role, rendering the interactions between the carbon chain and the phenyl group a structural determinant when all 1^st^ generation amino groups are neutral.

## Conclusion

In this work, we performed a full pH titration of the diaminopyrrolic tripodal receptor and a detailed analysis of the conformational dependence of this molecule with pH. We also studied the interaction between the receptor and the mannoside. Our results show that there is no significant difference in the conformational space of the receptor in ACN and in water (at pH > 4.0). Interestingly, in the presence of the mannoside, the number of hydrogen bonds between the two molecules is significantly larger in ACN than in water. This is probably the reason that the receptor is able to be selective towards mannoside in the organic solvent. There are several works, both theoretical [38–40] and experimental [1–6,37,41–42], showing that the interaction between sugars and their receptors is mainly driven by hydrogen bonds. In fact, there are several experimental structures of mannosides similar to the one used in this work in the protein data bank that also show a significant hydrogen bond network stabilizing the interaction between the sugar molecule and the protein (PDB IDs: 4avi [41], 1tr7 [42], 1uwf [42]). These observations indicate that the cause hindering the receptor to be active in water lies in the incapacity to form sufficient hydrogen bonds with the mannoside. Probably, the high propensity to form hydrogen bonds with water and the high flexibility of the arms of the receptor, are the main reasons that explain its low binding affinity in aqueous solvent.

This work also presents a new application of the stochastic titration constant-pH molecular dynamics method to a synthetic receptor for sugars. This method was able to describe the protonation/conformation coupling by correctly sample the occupation of titrable residues at the desired pH value.

## Experimental

### Simulations setup

As indicated in the introduction, two sets of simulations were performed (Table 1): A set of constant-pH MD simulations at 10 pH values (3 replicates of 100 ns each) and a set of MM/MD simulations in both water (at different protonation states) and acetonitrile (10 replicates of 100 ns each). The choice of protonation states was based on the pH-dependent proton distribution in the receptor from the constant-pH MD simulations. The fully deprotonated state (W^q0^) is typical of a high pH value (~10), the two states with 3 protons (W^q3a^ and W^q3b^) are typical of intermediate pH values (~4–7), and the 4 protons state (W^q4^) is representative of pH 3.0. This pH assignment to the protonation states was done according to the population analysis obtained in the constant-pH MD simulations (see Supporting Information File 1).

**Table 1:** Summary of the simulations used in this work. All replicates were run for 100 ns.

Solute	Simulation method	Solvent	Protonation state	pH	Replicates

receptor	Constant-pH MD	Water (1268 molecules)	NA	1, 2, 3, 4, 5, 6, 7, 8, 9 and 10	3 per pH

		ACN (535 molecules)	Fully deprotonated		3
		
receptor + mannoside	MD	Water (1926 molecules)	Fully deprotonated (W^q0^)	NA	10

			3 protons in 2^nd^ generation (W^q3a^)		

			2 protons in 2^nd^ generation and 1 in 1^st^ (W^q3b^)		

			3 protons in 2^nd^ generation and 1 in 1^st^ (W^q4^)		

		ACN (612 molecules)	Fully deprotonated		

### Constant-pH MD settings

All constant-pH MD simulations were performed using the stochastic titration constant-pH MD method implemented for the GROMACS package, developed by Baptista et al. [15,21]. These simulations ran following a stop-and-go procedure that alters between a protonation state calculation where the new states are obtained from a Monte Carlo run using Poisson–Boltzmann derived energy terms and two MM/MD runs (one with the solute frozen to allow it to adapt to the new protonation states – 0.2 ps and another of the unconstrained system – 2.0 ps). All six amino groups were titrated at all simulated pH values.

#### MM/MD settings (MM/MD simulations and MM/MD part of constant-pH MD simulations)

The MM/MD simulations were performed using GROMACS 4.0.7 [43–44] and adapted parameters from the GROMOS 56A_CARBO_ [45–46] force field. In all the constant protonation simulations, the neutral secondary amino groups were built in the R configuration. In the constant-pH MD simulations, the chirality of the amino groups is not important due to the continuous exchange of protons. A time step of 2 fs was used in the leap-frog algorithm. A rhombic dodecahedral simulation box with periodic boundary conditions was used. The non-bonded interactions were treated using a twin-range cutoff of 0.8/1.4 nm and the neighbor lists were updated every 5 steps (10 fs). Electrostatic long range interactions were treated with a generalized reaction field [47] with an ionic strength of 0.1 M and a relative dielectric constant of 54 for systems with water [48] and 35.84 for systems solvated with acetonitrile [49]. The v-rescale temperature coupling was used (298.0 K) with a coupling constant of 0.1 with solvent and solute separately coupled to the bath. The Berendsen coupling bath [50] was used to treat pressure (1 bar) with coupling constant 0.5. Isothermal compressibility of 4.50 × 10^−5^ bar^−1^ for water and 8.17 × 10^−5^ bar^−1^ for acetonitrile [51–52] were used. All bonds were constrained using the P-LINCS algorithm [53].

The energy minimization steps were performed using a combination of both steepest descent and limited-memory Broyden–Fletcher–Goldfarb–Shanno methods. The initiation was performed in 3 steps of 100 ps, 200 ps, and 200 ps with different restraints.

In the receptor + mannoside simulations, a distance restraint of 100 kJ mol^−1^ nm^−2^ was used between all pairs of atoms in the two rings (6 in substituted phenyl group in the receptor and 6 in the sugar ring of the mannoside). Hence, a penalty was added when the distance between any pair of atoms in the two rings exceeded 1.0 nm. The used function for this energy penalty is quadratic between 1.0 and 1.2 nm and linear above 1.2 nm. This restraint creates a sphere around the receptor that maintains the two molecules close to each other. It is important to mention that within this sphere, no unphysical restrain forces are applied to the interacting molecules.

#### PB/MC settings

The PB/MC calculations were done as previously described [26]. The MEAD 2.2.9 [25] software package was used for PB calculations performed with the atomic charges and radii taken from the MM/MD force field. The model compound p*K*_a_ value used (10.64 for all equivalent amino groups) is the p*K*_a_ of the dimethylamine [54]. It was used a dielectric constant of 2 for the solute and 80 for the solvent. Grid spacing of 0.1 nm, in the coarser calculation, and 0.025 nm, in the focusing procedure, were used in the finite difference method [55]. The molecular surface was determined using a rolling probe of 0.14 nm and the Stern layer was 0.2 nm. The temperature used was 298 K and the ionic strength was 0.1 M.

The MC calculations were performed using the PETIT (version 1.5) [56] software with 10^5^ steps for each calculation. Each step consisted of a cycle of random choices of protonation state (including tautomeric forms) for all individual sites and for pairs of sites with a coupling above 2.0 p*K*_a_ units [56–57], followed by the acceptance/rejection step according to Metropolis criterion [58]. The last protonation state is then used for the MM/MD part.

#### Analyses

The last 90 ns of each simulation were used for analyses. Several tools from the GROMACS software package [43–44] were used and others were developed in-house. The RMSD calculations followed a previously published procedure [59] that takes into account the symmetry of the receptor. The PyMOL 0.99RC6 software (http://www.pymol.org) was used to obtain rendered conformational images.

Energy landscapes were obtained from different 2D spaces by computing kernel estimates of the data probability densities [60] on grids of 2 pm^2^ bins, using a Gaussian kernel. The probability density surface was then converted to an energy surface according to:


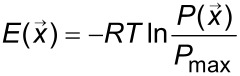


where 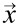 is a coordinate in a 2D space and *P*_max_ is the maximum of the probability density function, 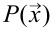.

The calculations of correlation-corrected errors for averages were computed using standard methods based on the auto-correlation function of the property measured to determine the number of independent blocks in the simulations [61].

## Supporting Information

File 1 Diagrams with the population of each microstate, histograms of *R*_g_, free energy profiles and schematic representation of the position of the arms at all pH values.
