# Supplementary material for: Why a diaminopyrrolic tripodal receptor binds mannosides in acetonitrile but not in water?
Source: Beilstein J Org Chem. 2014 Jul 3;10:1513–23. doi: 10.3762/bjoc.10.156 (PMC4142876; doi:10.3762/bjoc.10.156)

SUPPORTING INFORMATION

for

Why a diaminopyrrolic tripodal receptor binds mannosides in acetonitrile but not in water?

Diogo Vila-Viçosa[1], Oscar Francesconi[2] and Miguel Machuqueiro[1]*

Address: [1]Centro de Química e Bioquímica, Departamento de Química e Bioquímica, Faculdade de Ciências, Universidade de Lisboa, 1749-016 Lisboa, Portugal, Phone: +351-21-7500112. Fax: +351-21-7500088 and [2]Dipartimento di Chimica, Università di Firenze, Polo Scientifico e Tecnológico, 50019 Sesto Fiorentino, Firenze, Italy

Email: Miguel Machuqueiro* - machuque@fc.ul.pt

*Corresponding author

Diagrams representing the population of all microstates at all simulated pH values

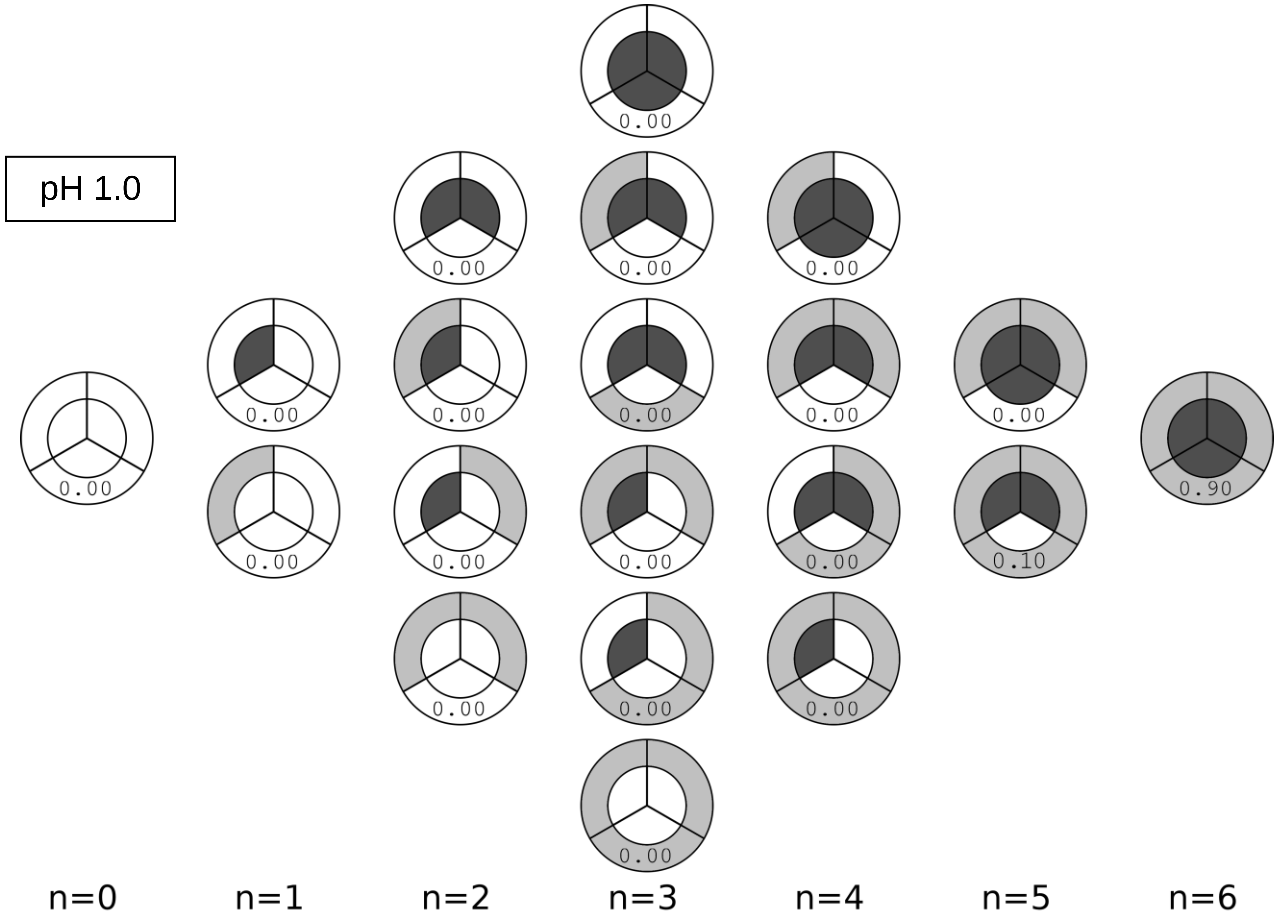

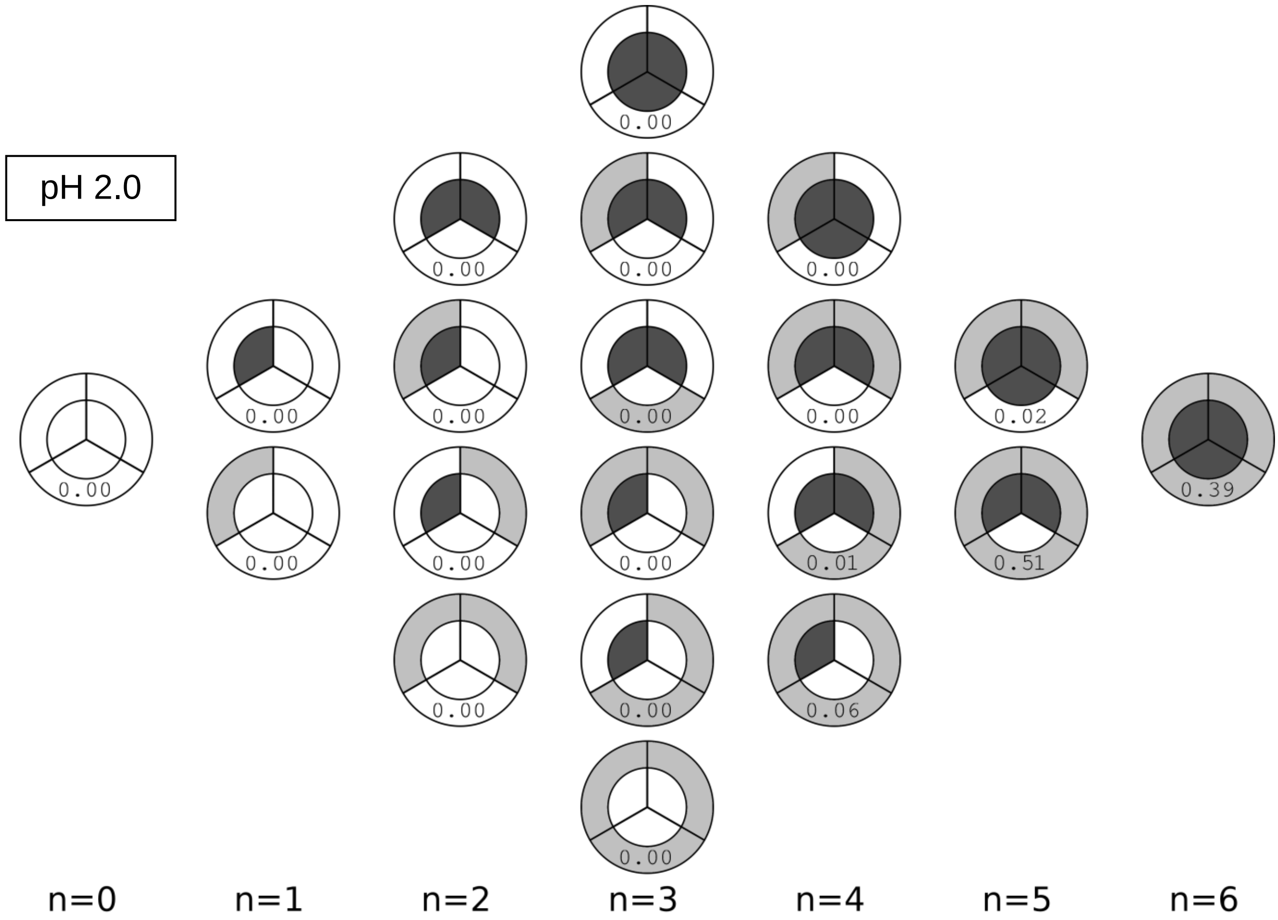

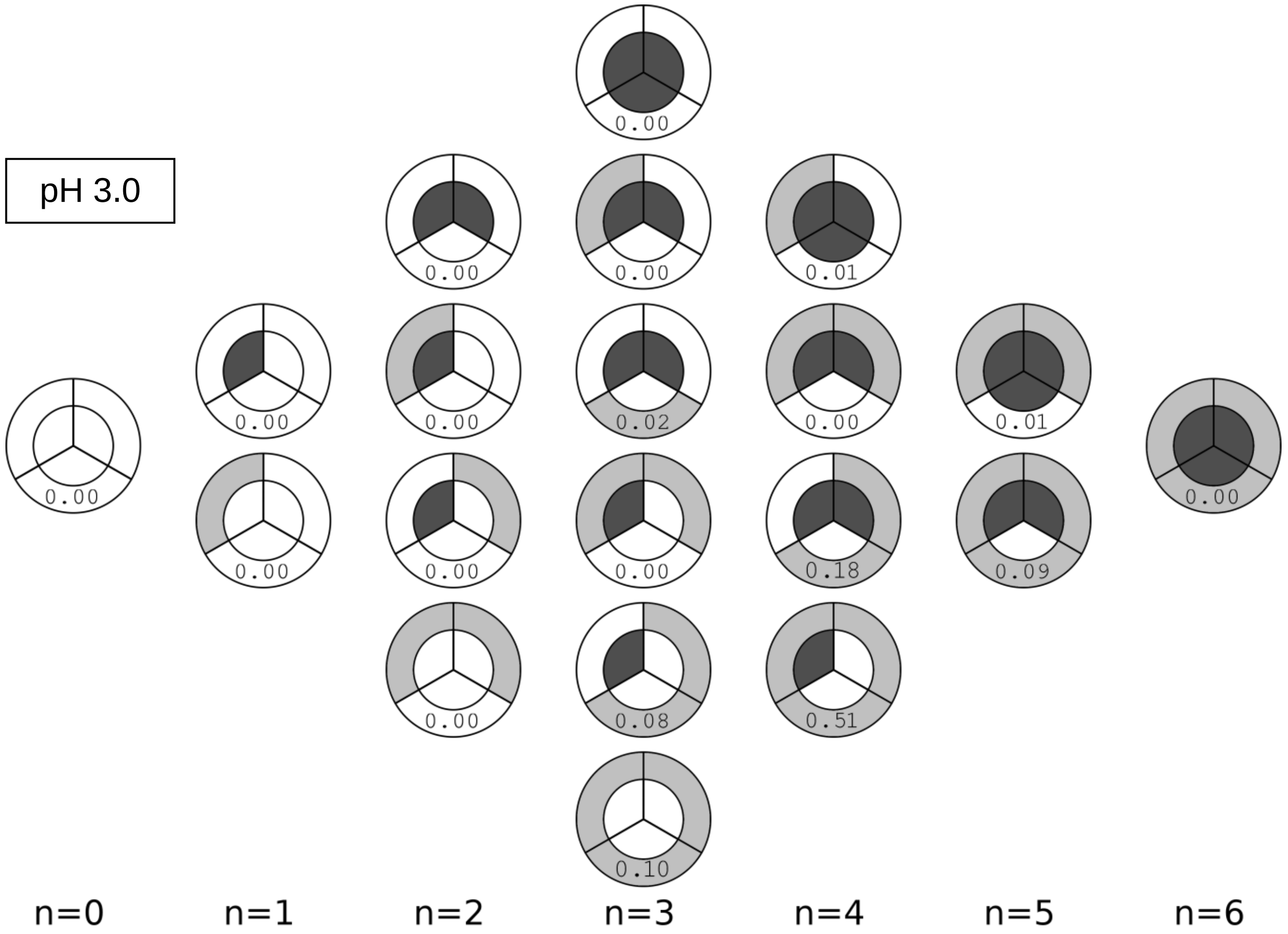
pH 3.0
0.00
0.00
0.00
0.00
0.00
0.00
0.00
0.00
0.00
0.02
0.00
0.08
0.10
0.01
0.00
0.18
0.51
0.01
0.09
0.00
n=0
n=1
n=2
n=3
n=4
n=5
n=6

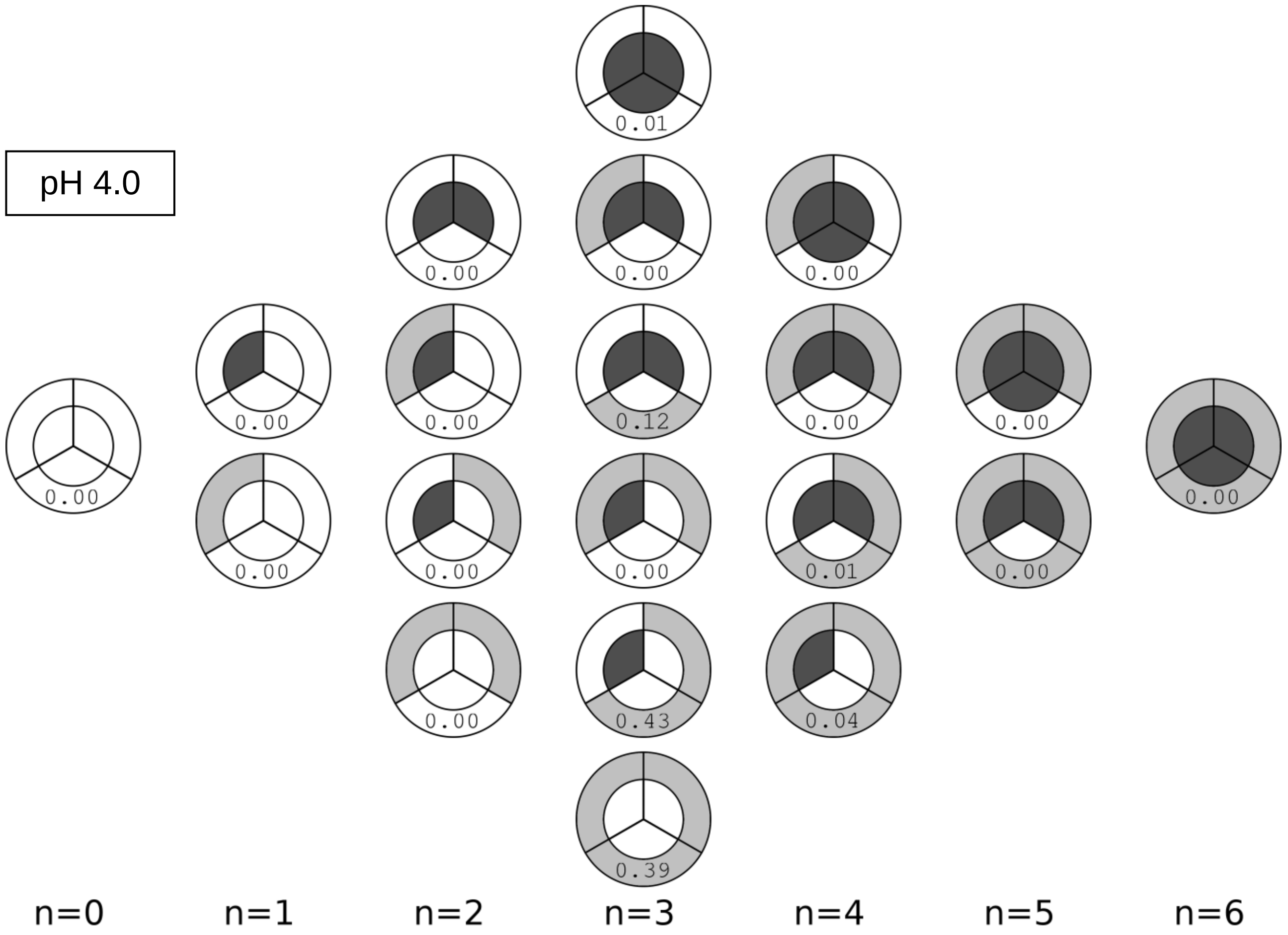
pH 4.0
0.00
0.00
0.00
0.00
0.00
0.00
0.00
0.01
0.00
0.12
0.00
0.43
0.39
0.00
0.00
0.01
0.04
0.00
0.00
0.00
n=0
n=1
n=2
n=3
n=4
n=5
n=6

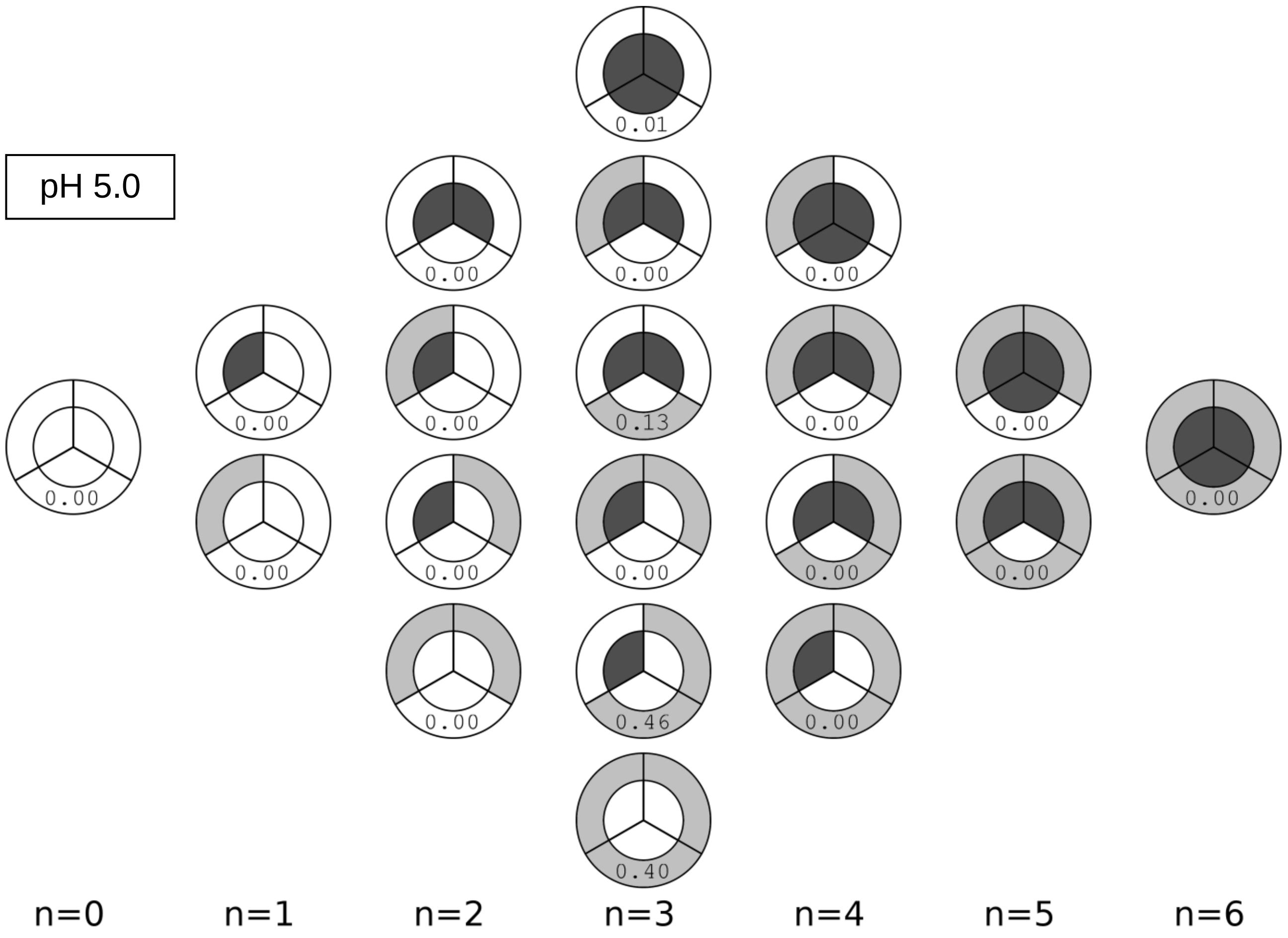
pH 5.0
0.00
0.00
0.00
0.00
0.00
0.00
0.00
0.01
0.00
0.13
0.00
0.46
0.40
0.00
0.00
0.00
0.00
0.00
0.00
0.00
n=0
n=1
n=2
n=3
n=4
n=5
n=6

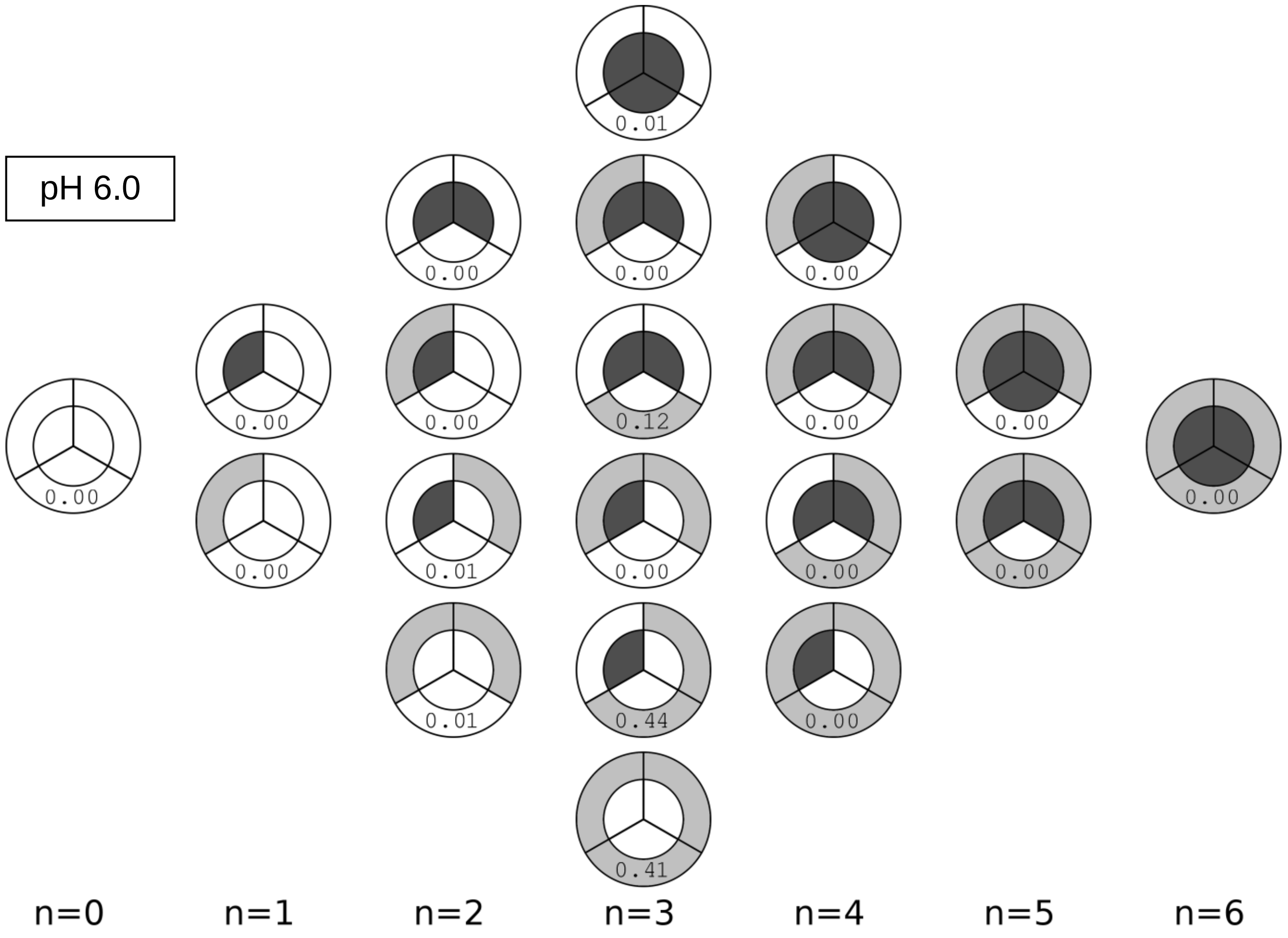
pH 6.0
0.00
0.00
0.00
0.00
0.00
0.01
0.01
0.01
0.00
0.12
0.00
0.44
0.41
0.00
0.00
0.00
0.00
0.00
0.00
0.00
n=0
n=1
n=2
n=3
n=4
n=5
n=6

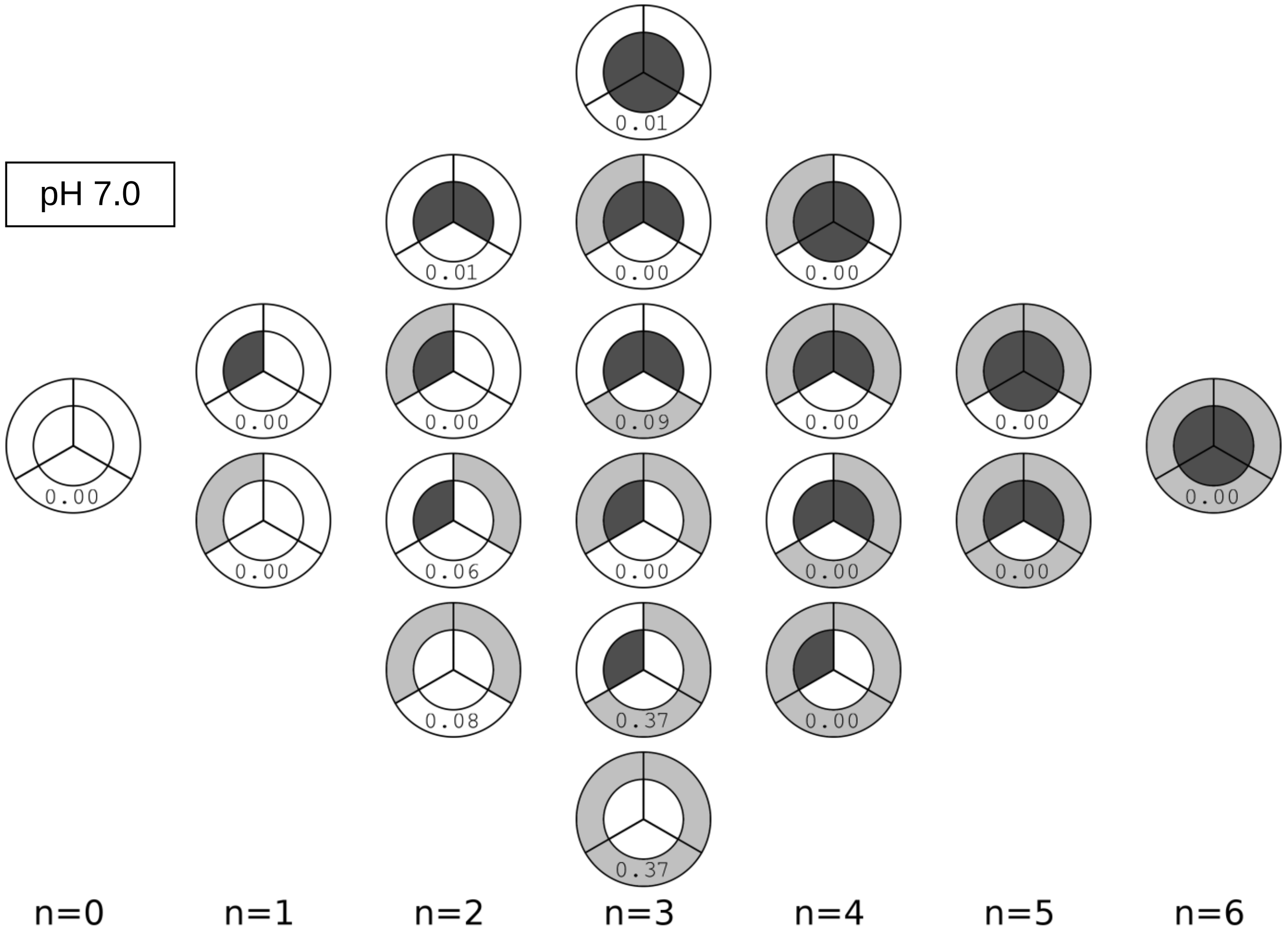
pH 7.0
0.00
0.00
0.00
0.01
0.00
0.06
0.08
0.01
0.00
0.09
0.00
0.37
0.37
0.00
0.00
0.00
0.00
0.00
0.00
0.00
n=0
n=1
n=2
n=3
n=4
n=5
n=6

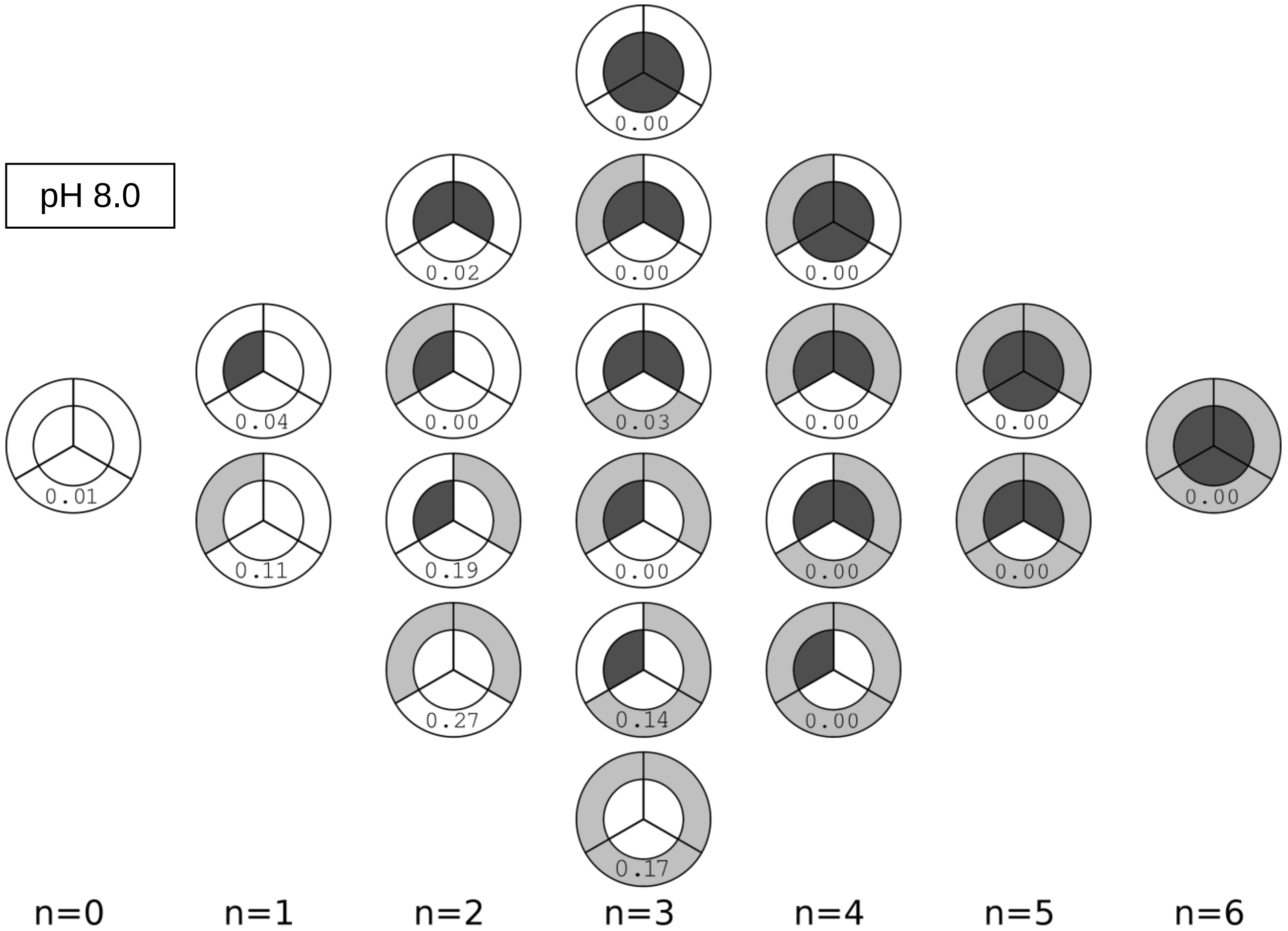
pH 8.0
0.01
0.04
0.11
0.02
0.00
0.19
0.27
0.00
0.00
0.03
0.00
0.14
0.17
0.00
0.00
0.00
0.00
0.00
0.00
0.00
n=0
n=1
n=2
n=3
n=4
n=5
n=6

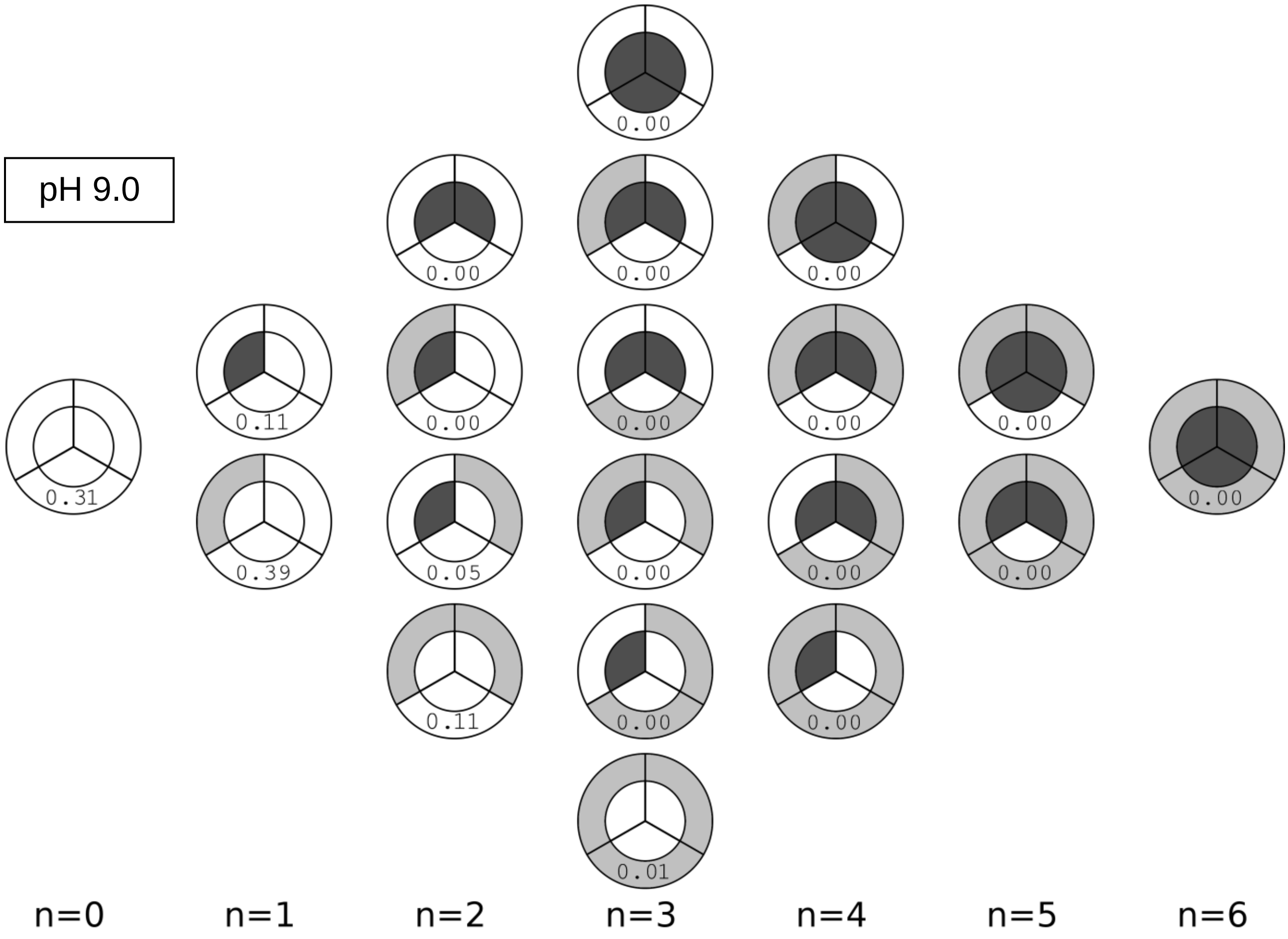
pH 9.0
0.31
0.11
0.39
0.00
0.00
0.05
0.11
0.00
0.00
0.00
0.00
0.00
0.00
0.01
0.00
0.00
0.00
0.00
0.00
0.00
0.00
n=0
n=1
n=2
n=3
n=4
n=5
n=6

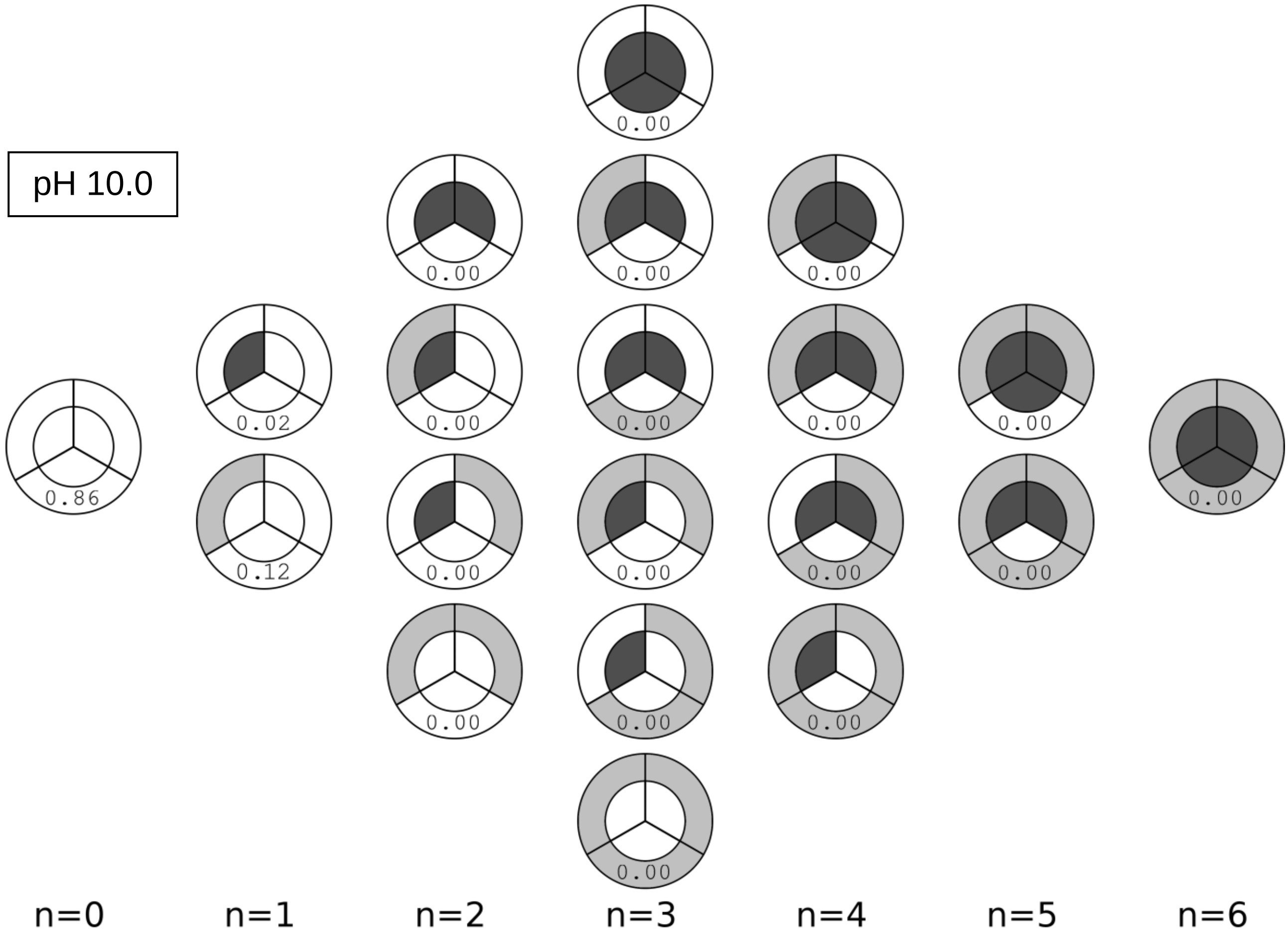
pH 10.0
0.86
0.02
0.12
0.00
0.00
0.00
0.00
0.00
0.00
0.00
0.00
0.00
0.00
0.00
0.00
0.00
0.00
0.00
0.00
0.00
0.00
n=0
n=1
n=2
n=3
n=4
n=5
n=6

Radius of gyration (R_g) histograms for the receptor at all simulated pH values and acetonitrile (ACN)

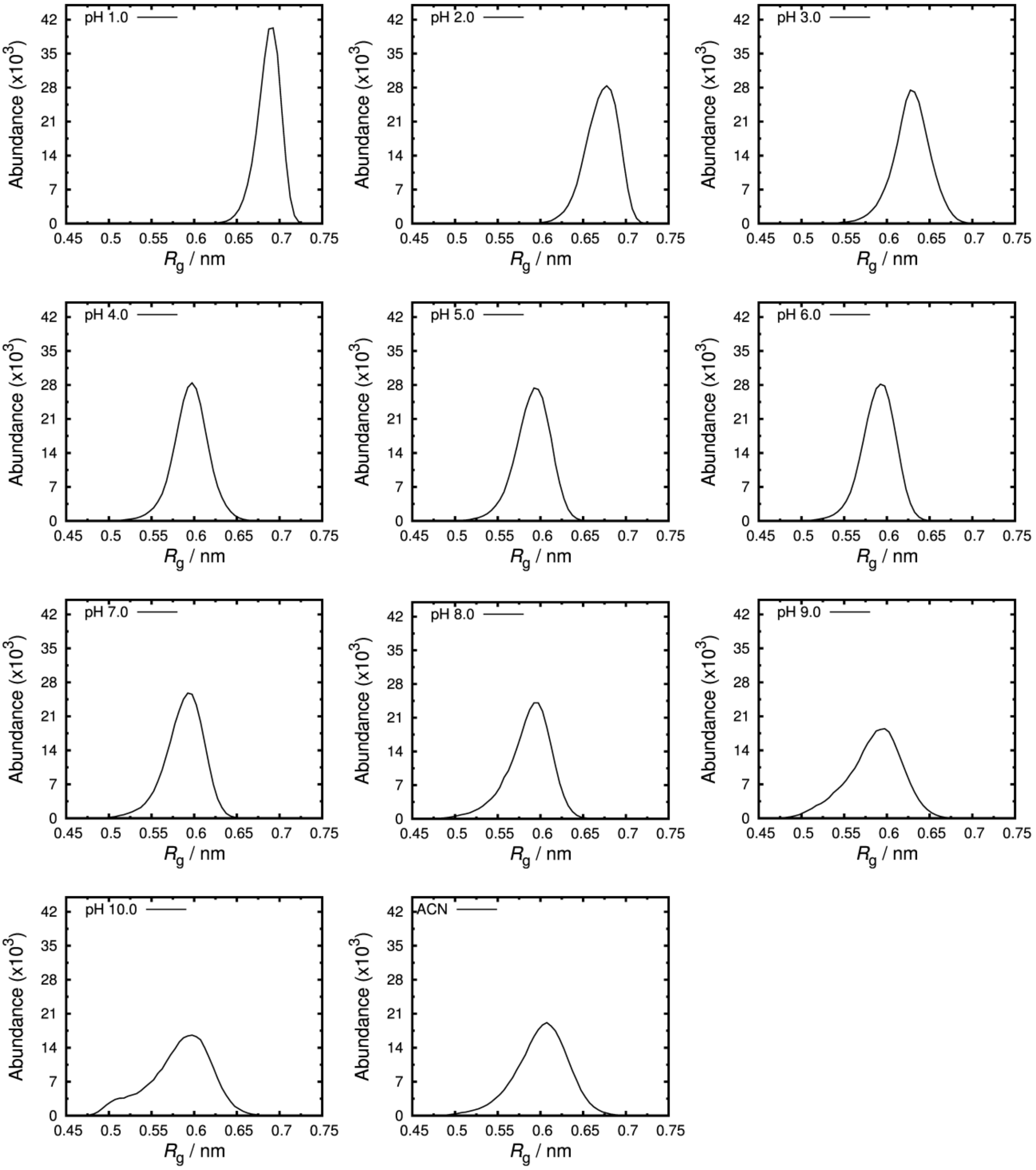

Free energy profiles for the receptor at all simulated pH values and ACN using RMSD and R_g as structural coordinates

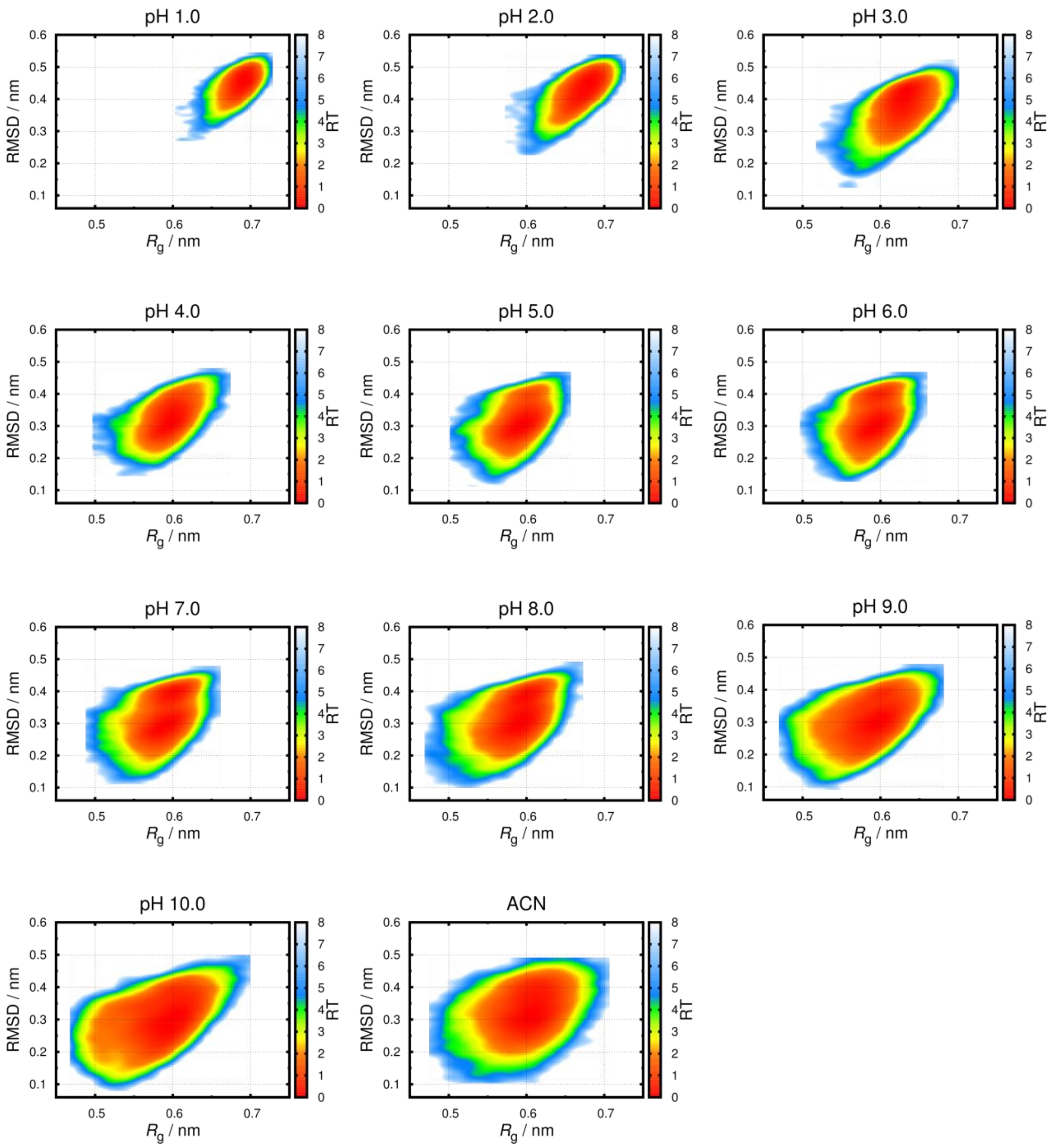

Schematic representation of the position of the arms of the receptor. Position of nitrogen atoms of the pyrrolic rings relatively to the phenyl ring in 900 conformations at all simulated pH values and ACN.

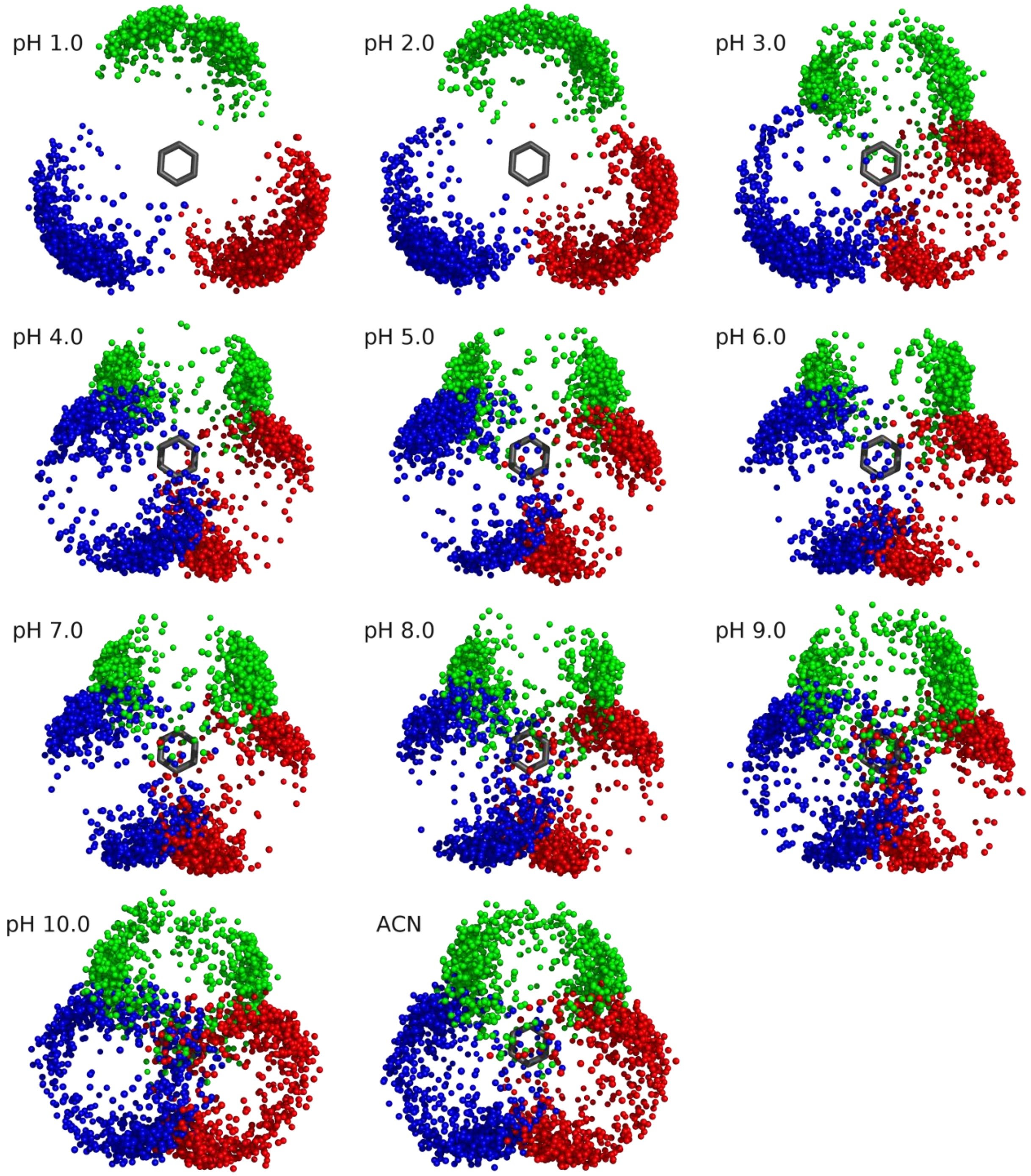

Supplement: File 1 — Diagrams with the population of each microstate, histograms of Rg, free energy profiles and schematic representation of the position of the arms at all pH values. [file Beilstein_J_Org_Chem-10-1513-s001.pdf]
